# Erzhu Jiedu decoction ameliorates liver precancerous lesions in a rat model of liver cancer

**DOI:** 10.7150/jca.49554

**Published:** 2020-10-22

**Authors:** Yang Cheng, Tianyang Chen, Jianjie Chen

**Affiliations:** 1 Department of liver disease, Hospital for Infectious Diseases of Pudong District, Shanghai 201299, P.R. China; 2 Institute of liver disease, Shuguang Hospital affiliated to Shanghai University of Traditional Chinese Medicine, Shanghai 201203, P.R. China

**Keywords:** Erzhu Jiedu Decoction, Hepatocellular Carcinoma, Diethylnitrosamine, Liver Precancerous lesions, Traditional Chinese medicine

## Abstract

Precancerous lesions are the intermediate stage in the development of liver cancer from cirrhosis. Early intervention measures can effectively prevent the occurrence of liver cancer and prolong the lives of patients, resulting in greater economic effects. Erzhu Jiedu decoction (EJD) is a semiempirical formula that is used in the treatment of cirrhosis and liver cancer according to the academic philosophy of “Preventive treatment of disease” and has achieved good curative effects in clinical practice. The purpose of this study was to investigate the effect of EJD on liver precancerous lesions induced by diethylnitrosamine (DEN) in rats. The results showed that EJD improved the general conditions (body weight, ALT, AST, and GGT) and reduced the number of precancerous lesions in the rat model. Notably, the medium dose of EJD (1.05 g/kg) had better treatment effects than the low dose of EJD, and the high dose of EJD did not further improve the liver lesions compared to the medium dose of EJD. Moreover, EJD effectively reduced the DEN-induced GST-Pi, AFP, CK19, c-Myc, and Ki67 protein expression in liver precancerous tissues. Interestingly, EJD significantly reduced YAP and TAZ mRNA expression in the liver precancerous lesions. Collectively, EJD protects against in the initiation of liver cancer and the regulation of c-Myc and Hippo signaling pathways may be the underlying mechanism.

## Introduction

Primary liver cancer includes a heterogeneous group of malignancies with different histological characteristics and poor prognosis. According to the International Agency for Research on Cancer, liver cancer is the fifth most common cancer among men worldwide (523,000 cases per year, accounting for 7.9% of all cancers) and the seventh most common cancer among women (226,000 cases annually, accounting for 6.5% of all cancers). Hepatocellular carcinoma (HCC) is the major type of liver cancer. The main risk factors for HCC include viral infection, fatty liver disease, autoimmune liver disease, and chronic cholestasis liver disease 1. The incidence of HCC mainly occurs in developing countries and is closely related to age, gender and ethnicity. The onset of HCC is often overlooked because of the lack of typical clinical symptoms early in disease. Once a pathological examination confirms HCC, the opportunity for the best treatment effect has often been missed, and the prognosis is very poor.

In recent years, with the continuous advances in modern medical technology and the comprehensive use of various treatment strategies, the treatment methods for HCC have been continuously updated and enriched; these treatment methods include 1) nondrug therapy, such as surgical resection, radiofrequency ablation, radiotherapy, and liver transplantation, and 2) drug therapy, such assorafenib, cabotinib, and nivolumab, which significantly improves the prognosis of HCC patients, but the overall survival rate is still unsatisfactory 2, 3. Therefore, it is very important to explore the ideal drugs and methods for the treatment of HCC.

Precancerous lesions are mature histopathological precancerous tissue that have the highest risk of tumorigenesis and that develop preferentially due to activation of DNA damage checkpoints and persistent inflammation 4. In the past few years, precancerous lesions have been observed in a variety of tumors, including but not limited to colorectal cancer 5, pancreatic cancer 6, gastric cancer 7, and liver cancer 8, 9. Precancerous lesions of the liver represent a progression from quantitative change to qualitative change. The presence of precancerous lesions is an intermediate stage in the process of initiation, promotion and evolution in the development of cancer 10. Some scholars believe that this process can be reversed. However, the basic pathological and physiological mechanisms that lead to precancerous liver lesions warrant further investigation. Importantly, intervention measures that reverse or interrupt the pathological process of liver cancer precancerous lesions may effectively inhibit the occurrence and development of liver cancer and extend the lives of patients, thereby resulting in greater economic benefits.

At present, there are still no ideal drugs or methods for the treatment of precancerous liver lesions. Notably, traditional Chinese medicine (TCM) has unique advantages in this field 11-13. Chinese herbs and extracts, such as Ginkgo biloba extract 14, Resveratrol 15 and moxibustion 16 have made great progress in inhibiting liver precancerous lesions. Erzhu Jiedu decoction (EJD) is a traditional Chinese medicine formula that is widely used to treat cirrhosis and liver cancer. Previously, we explored the clinical efficacy of EJD in the treatment of hepatitis B cirrhosis with hyper-alpha-fetoproteinemia. Seventy-two patients with hepatitis B cirrhosis and high alpha-fetoproteinemia who met the inclusion criteria were randomly divided into an EJD treatment group and a control group. After 48 weeks of treatment, alpha-fetoprotein (AFP), alpha-fetoprotein heterogeneity (AFP-L3), serum alanine aminotransferase (ALT), aspartate radon aminotransferase (AST), total bilirubin (TBil), serum glutamyl transpeptidase (GGT), serum albumin (Alb) levels were significantly improved by EJD treatment compared with the control group (Article in Chinese). Thus, EJD exhibited great beneficial effect in clinical practice. However, the effects of EJD on liver precancerous lesions and the precise mechanisms are not known.

In this study, diethylnitrosamine (DEN) was used to establish a rat model of liver precancerous lesions, and EJD was used as preventive intervention. The general conditions of the rats in each group, the changes in liver pathology, and the changes in biochemical indicators were analyzed. Immunohistochemical staining was used to assess the indicators of the occurrence and progression of liver precancerous lesions. In addition, the underlying molecular mechanism was preliminarily explored. In this study, we aimed to provide a basis for further development of effective Chinese medicine agreement parties for the prevention and treatment of liver precancerous lesions, and lay the foundation for future clinical research.

## Materials and methods

### Experimental drugs

EJD was provided by Shuguang Hospital Affiliated to Shanghai University of Traditional Chinese Medicine. EJD consisted of the following six original medical herbs: *Atractylodis Rhizoma* (3 g), *Atractylodes macrocephala Koidz* (3 g), *Scutellaria barbarta D.Don* (1 g), *Hedyotis diffusa Willd* (1 g), *Radix salvia miltiorrhizae* (2 g), and *Carapax Trionycis* (2 g). All the original herbs were produced in China and provided by Chinese Medicine Pharmacy of Shuguang Hospital. Herbs were mixed and soaked in 4 volumes of water, boiled twice for 30 min, extracted 3 times, and concentrated to generate EJD. Subsequently, EJD was dissolved in 0.9% saline at the indicated concentrations for later use. Additionally, EJD needs to be ready to use. Cinobufotalin capsules were purchased from Shaanxi Dongtai Pharmaceutical Co., Ltd. (Shanxi, China). DEN was obtained from Sigma-Aldrich (N0756, Shanghai, China).

### Animals

Male Wistar rats (6-week-old, 180 ± 10 g) were purchased from Bikai Laboratory Animal Co., Ltd (Shanghai, China; license number: SCXK (Shanghai) 2013-0016) and maintained under specific pathogen-free conditions. All animals were fed a standard diet with controlled condition (temperature, 25 °C ± 1 °C; humidity, 50%) with free access to food and water. The animal experimental protocols were approved by the Ethics Committee of Shanghai University of Traditional Chinese Medicine. All experimental procedures were performed according to the guidelines of the Care and Use of Laboratory Animals by the National Institute of Health, China.

### Model generation, grouping, and drug treatment

After one week of acclimatization, the rats were randomly divided into the following six groups: control+vehicle (n = 8), DEN+ vehicle (n = 14), DEN + cinobufotalin (n = 10), DEN + low-dose EJD (n = 10), DEN + medium-dose EJD (n = 10), and DEN + high-dose EJD (n = 10) groups. The rats in the control + vehicle group were given an intraperitoneal injection of normal saline and received intragastric administration. To establish a rat model of liver cancer, the rats in the other groups were intraperitoneally injected with DEN (50 mg/kg) 2 times a week for 4 consecutive weeks, followed by one time a week from week 5-14. The rats in the three EJD groups were administered with 0.525 g/kg (low dose), 1.05 g/kg (middle dose) or 2.1 g/kg (high dose) of EJD via gastric perfusion. The doses of EJD in rat model were used based on the daily dose taken by adults in our hospital. The maximum concentration was identified according to the maximum nontoxic dose in our preliminary experiment. The dose of DEN (50 mg/kg) used to generate the rat model was reported elsewhere. The rats in the cinobufotalin group were administered with 0.15 g/kg cinobufotalin. During the experiment, the general condition of each rat was observed. The body weight of the rats was recorded weekly.

### Liver index and spleen index

Rat liver index and spleen index were calculated as follows: liver index = liver wet weight (g) / body weight (g) × 100%; spleen index = spleen wet weight (g) / body weight (g) × 100%.

### Measurement of serum ALT, AST, and GGT

The obtained blood samples were kept at room temperature for 1 h. Serum samples were then collected after centrifugation at 1000 × g for 15 min. The plasma alanine amino transferase (ALT), aspartate amino transaminase (AST), and glutamyl transpeptidase (GGT) activity was analyzed by a commercial kit purchased from Rsbio (Shanghai, China) in accordance to the manufacturer's instructions.

### Hematoxylin and eosin staining

Rat liver tissues were fixed in 10% formalin, embedded in paraffin, and cryosectioned into 5 μm sections. After deparaffinization and rehydration, the sections were stained with hematoxylin and eosin (H&E) routinely. Finally, the liver specimens were evaluated by light microscopy.

### Immunohistochemistry

The liver sections were dewaxed and rehydrated, followed by antigen retrieving. Then, 3% H_2_O_2_ in methanol was added to quench endogenous peroxidase activity. Subsequently, the sections were blocked with 10% BSA (Sangon, Shanghai, China). After that, slides were incubated with primary antibodies: GST-Pi (dilution 1:200, Abcam, ab53943), AFP (dilution 1:300, Cell Signaling Technology, #4448), CK19 (dilution 1:200, Proteintech, 10712-1-AP), c-Myc (dilution 1:200, Abcam, ab32072), and Ki67 (dilution 1:1000, Cell Signaling Technology, #9449) at 4°C overnight. The next day, the sections were incubated with a horseradish peroxidase-conjugated secondary antibody IgG at room temperature for 45 min.

Finally, positive staining was visualized with diaminobenzidine substrate liquid (Gene Tech, Shanghai, China), and counterstained with hematoxylin. All the sections were observed and photographed with a microscope (Carl Zeiss, Germany). IPP6.0 software was used to analyze the expression of indicted proteins through measuring the average optical density from 3 randomly selected fields.

### Quantitative real-time PCR

Total RNA from normal liver and liver precancerous tissues was extracted using the TRIzol reagent (Takara, Japan) according to the manufacturers' instructions. The concentration of total RNA was determined by a NanoDrop 2000 spectrophotometer (Thermo Fisher Scientific Inc., USA) followed by reverse transcription (RT) using the a RT first-strand cDNA synthesis kit (Takara, Japan) for RT-PCR. Quantitative real-time PCR was performed with a SYBR Green Premix Ex Taq (Takara, Japan) in a 10 μL reaction system on a 7500 Real-time PCR system (Applied Biosystems, Inc. USA). The relative mRNA expression was calculated by normalizing to an internal control β-actin. Each reaction was performed in triplicate. The primers used in this study were shown in Table 1.

### Statistical analysis

Data were presented as the means ± SDs. The calculations were performed using GraphPad Prism version 4.03 for Windows (GraphPad Software Inc., San Diego, CA). The ANOVA followed by a post-hoc test was used for comparisons of between groups in this study. P < 0.05 was considered statistically significant.

## Results

### Basic information in DEN-induced liver precancerous lesions after treatment with EJD

**Figure 1:**
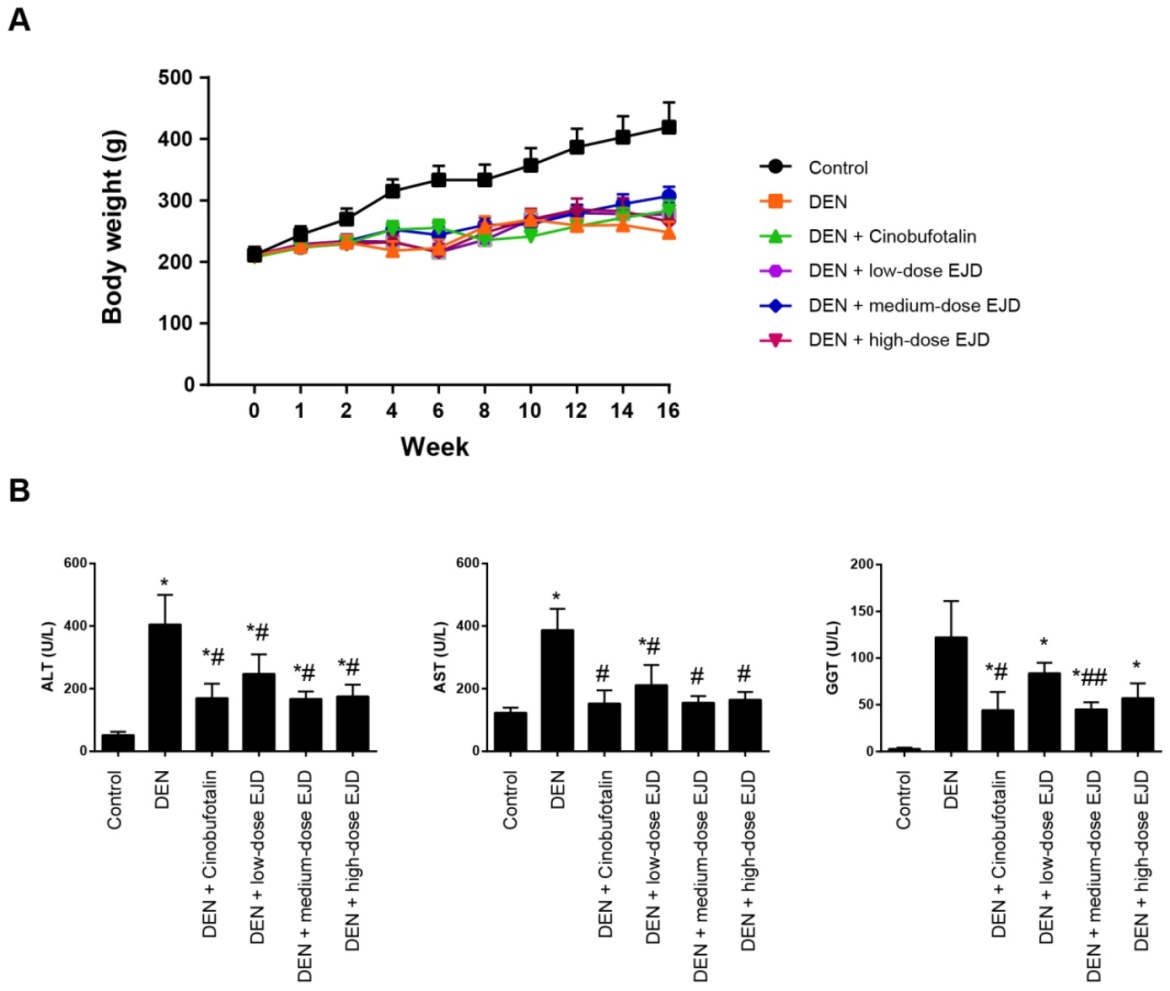
Body weight and liver function in a rat model of DEN-induced precancerous liverlesions after EJD treatment. (A) Body weights of the rats in the model group, the three EJD groups, and the cinobufotalin group. (B) Serum ALT, AST, and GGT activity in the DEN-induced precancerous liver lesion model treated with either EJD or cinobufotalin. * Represents comparison with the normal group, *p < 0.05; # represents comparison with the model group, #p < 0.05 and ##p < 0.01.

First, we explored the potential therapeutic efficacy of EJD in Wistar rats challenged with DEN. In this study, three test groups were designed with increasing doses of EJD, and cinobufotalin treatment was used as the positive control. Beginning in week 4, we observed significant vertical dorsal hair, slow movement, and listlessness in the model group, the three EJD groups and the cinobufotalin group. Compared with that in the model group, the final body weights of the rats in the three EJD groups and the cinobufotalin group was significantly increased; specifically, medium-dose EJD group exhibited the highest beneficial effect on DEN-induced rat model (**Fig. 1A**). Additionally, the increase in body weight induced by EJD was accompanied by a remarkable reduction in the serum level of alanine amino transferase (ALT), aspartate amino transaminase (AST), and glutamyl transpeptidase (GGT) (**Fig. 1B**).

### EJD inhibits the formation of liver precancerous lesion

**Figure 2:**
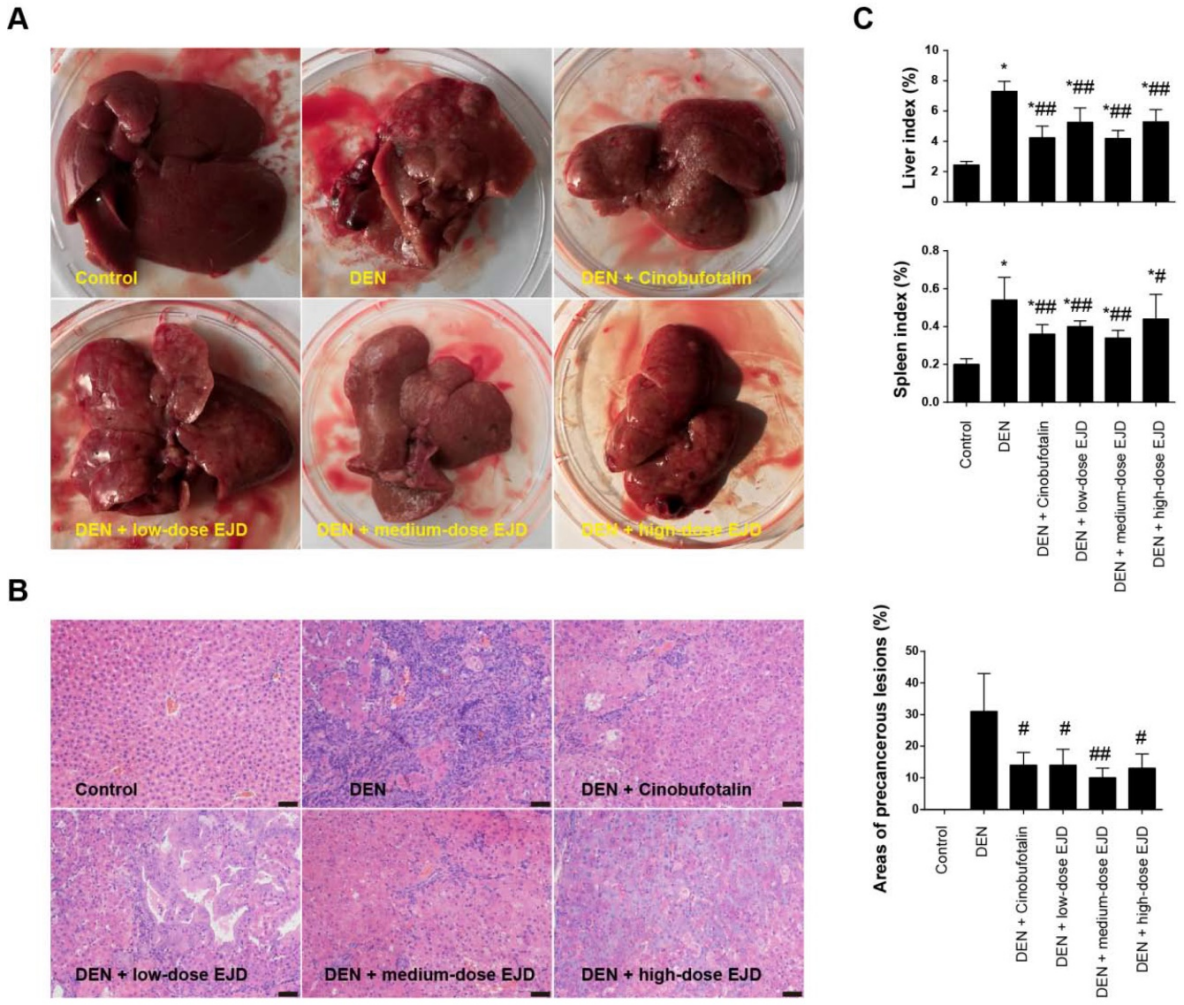
EJD inhibits precancerous liver lesions. (A) Photographs of the representative rat livers in the control group, model group, three EJD groups, and cinobufotalin group. (B) Hematoxylin & eosin staining of liver sections from the control group, model group, three EJD groups, and cinobufotalin group; magnification at 200×; scale bar, 50 μm. (C) Liver and spleen indexes in the control group, model group, three EJD groups, and cinobufotalin group. * Represents comparison with the normal group, *p < 0.05; # represents comparison with the model group, ^#^p < 0.05 and ^##^p < 0.01.

Examination of the liver morphology showed that the livers in the rats in the model group were dull, rough, and hard and exhibited a dark red color; in addition, the liver surfaces were changed due to the presence of single or widespread white tumor nodules of different sizes (**Fig. 2A**). In contrast, the livers of the rats in the EJD groups and the cinobufotalin group had dark lusters, hard textures, and fewer nodules on their surfaces (**Fig. 2A**). HE staining revealed that the hepatoctyes in model group exhibited different degrees of degeneration, necrosis, disordered arrangement, cellular pleomorphism, big nucleus, increased nuclear/cytoplasmic ratios, and cell dysplasia, which were accompanied by fibrous tissue growth and pseudolobule formation. Compared with those in the model group, the hepatocytes in the medium-dose EJD group had relatively normal morphology, and the extent of connective tissue hyperplasia was relatively low (**Fig. 2B**). Moreover, the liver index and spleen index of cinobufotalin group and the low-, medium- and high-dose groups of EJD were significantly lower than those of the model group (**Fig. 2C**). Collectively, these data above suggest that EJD exerts an inhibitory effect against liver precancerous lesion.

### EJD attenuates the expression of tumor biomarkers involved in liver precancerous lesion

**Figure 3:**
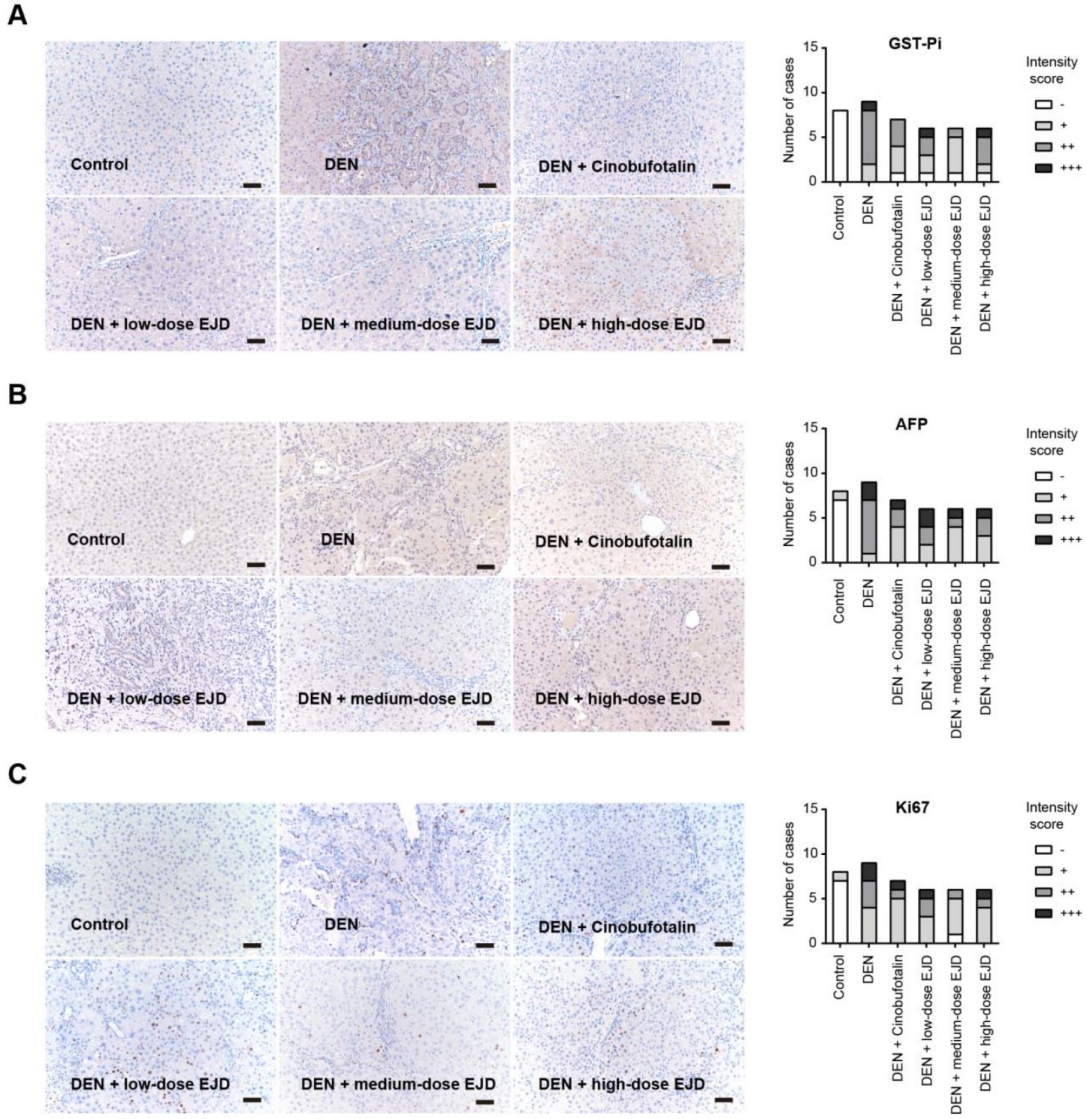
EJD reduces the expression of GST-Pi, AFP, and Ki-67 in a DEN-induced rat liver cancer model. Immunohistochemical analysis of GST-Pi (A), AFP (B), and Ki-67 (C) in the control group, model group, three EJD groups, and cinobufotalin group; magnification at 200×; scale bar, 50 μm.

Glutathione S-transferases (GSTs) are enzymes that catalyze the binding of glutathione (GSH) to a variety of hydrophobic and electrophilic compounds. The GST family contains many isozymes with a wide range of substrate specificities and these isozymes exist as homodimers and heterodimers of subunitsranging 17-28 kDain size. GST-Pi (GSTP1-1) is the most common and ubiquitous nonhepatic isoenzyme and is almost undetectable in normal hepatocytes, while is highly expressed in DEN-induced liver carcinogenesis. GST-Pi-positive lesions are widely considered as reliable and sensitive markers of liver cancer development 17, 18. By immunohistochemical analysis, we showed that GST-Pi expression in DEN-induced liver precancerous lesions was significantly attenuated by treatment with cinobufotalin and low-dose and medium-dose EJD; no significant improvement was observed after treatment with high-dose EJD (**Fig. 3A**).

Alpha-fetoprotein (AFP) is an acidic glycoprotein with a molecular weight of 69,000 Da that has been used as the "gold standard" diagnostic biomarker for HCC 19. Ki67 is a protein that is expressed in the nucleus only during cell division and acts as proliferation index for many types of cancers. Compared with that in the model group, the expressions of AFP and Ki67 were remarkably reduced in medium- and high-dose EJD groups (**Fig. 3B and 3C**).

**Figure 4:**
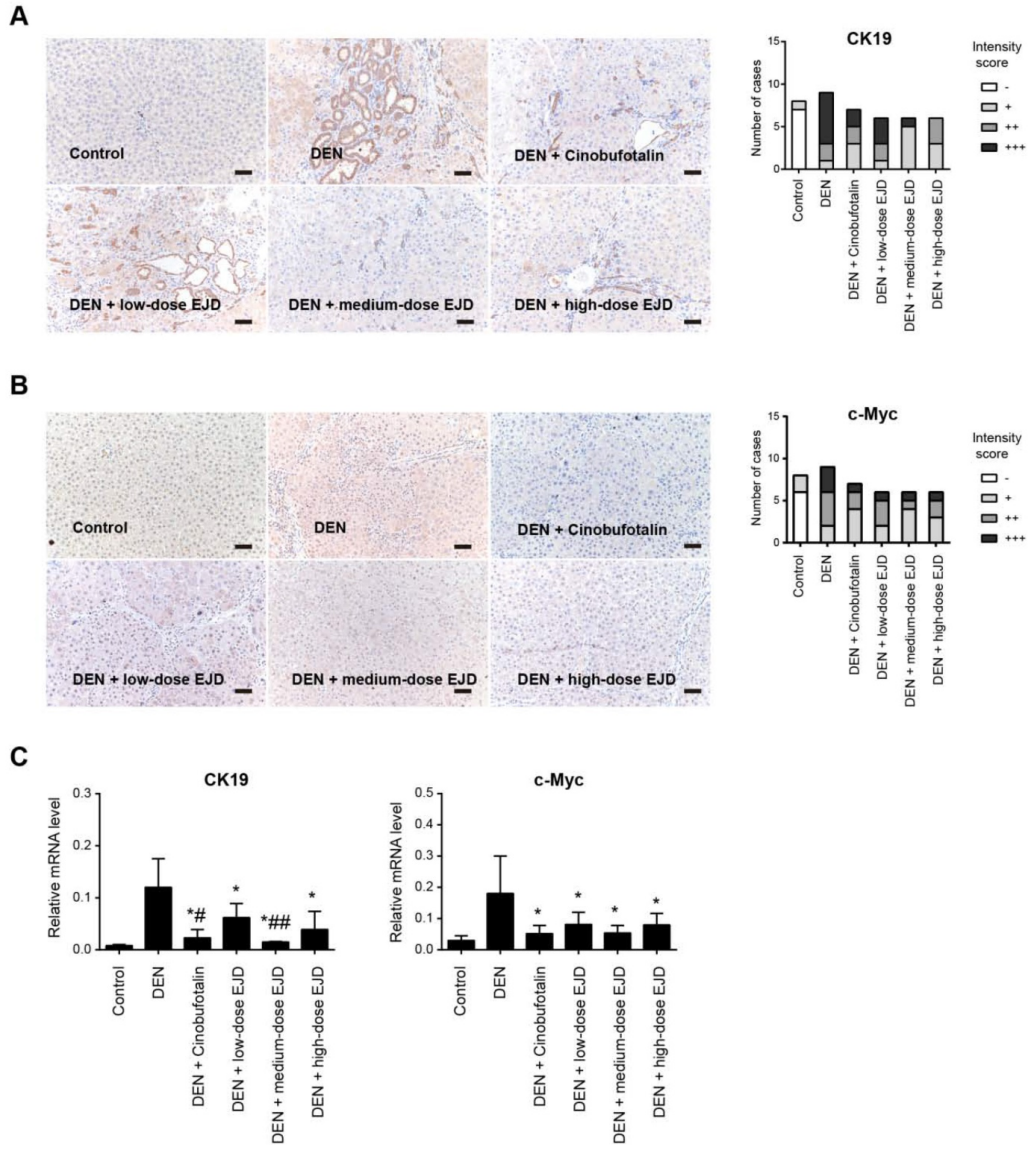
EJD reduces the expression of CK19 and c-Myc in a DEN-induced rat precancerous liver lesion model. Immunohistochemical analysis of CK19 (A) and c-Myc (B) in the control group, model group, three EJD groups, and cinobufotalin group; magnification at 200×; scale bar, 50 μm. (C) Real-time qPCR analysis of CK19 and c-Myc mRNA expression in the control group, model group, three EJD groups, and cinobufotalin group. * Represents comparison with the normal group, *p < 0.05; # represents comparison with the model group, ^#^p < 0.05 and ^##^p < 0.01.

Cytokeratin (CK) is an intermediate silk protein that is mainly expressed in epithelial cells. CK consists of type I (CK9-20) and type II (CK1-8) cytokeratins, which maintain the epithelial barrier and regulate innate immunity, intracellular signaling, cell adhesion, and epithelial cell proliferation and differentiation. CK19 is not expressed in normal liver cells, but is overexpressed during malignant transformation 20. The development of liver cancer is a multistep process of genetic changes that involves the activation of proto-oncogenes, which leads to a continuous increase in uncontrolled cell proliferation 21. C-Myc is an important proto-oncogene, that is involved in cell growth and cell differentiation, apoptosis, invasion, and many other biological activities; c-Myc overexpression leads to autonomous cell proliferation and is a crucial stimulus for the development of liver cancer 22. Compared with those in the normal group, CK19 and c-Myc protein were drastically increased in DEN-induced precancerous rat model group; the expression of CK19 and c-Myc proteins were reduced in the medium- and high-dose EJD groups compared to the model group (**Fig. 4A and 4B**). Moreover, real-time qPCR analysis showed that EJD inhibited CK19 and c-Myc expression in DEN-induced liver precancerous lesions (**Fig. 4C**). Collectively, these findings support a suppressive role of EJD in preventing liver precancerous lesion.

### EJD activates the Hippo signaling pathway in liver precancerous lesions

**Figure 5:**
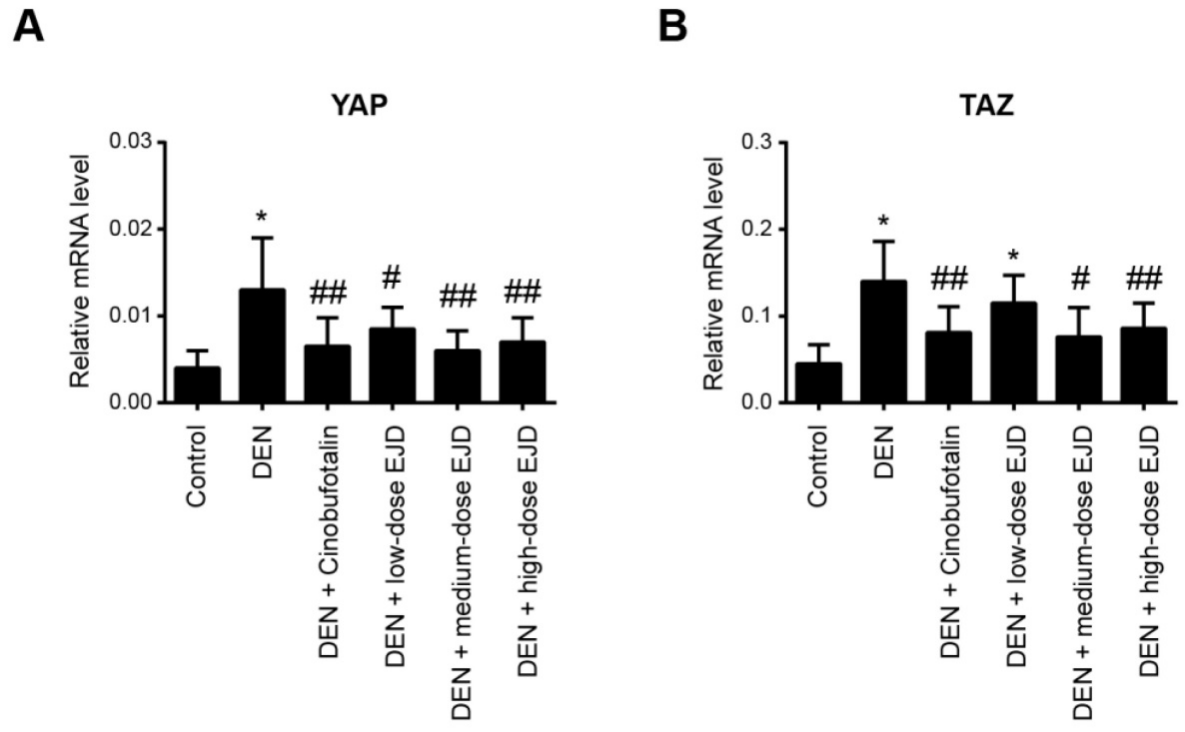
EJD activates the Hippo signaling pathway in liver precancerous lesions. Real-time qPCR analysis of YAP (A) and TAZ (B) mRNA expression in the control group, model group, three EJD groups, and cinobufotalin group. * Represents comparison with the normal group, *p < 0.05; # represents comparison with the model group, ^#^p < 0.05 and ^##^p < 0.01.

It has been well documented that the Hippo pathway regulates organ size and tissue homeostasis in different model organisms 23. The Hippo pathway is highly conserved under normal conditions and plays a role in inhibiting cancer development. YAP and TAZ are two closely related paralogs that mediate downstream effects of the Hippo pathway 24. By real-time qPCR analysis, we found that YAP and TAZ mRNA expression was significantly upregulated in the liver tissues with precancerous lesions; notably, cinobufotalin and the three different doses of EJD were sufficient to reduce the DEN-induced YAP and TAZ expression (**Fig. 5A and 5B**). Therefore, EJD might attenuate the formation of liver precancerous lesions by activation of the Hippo signaling pathway.

## Discussion

In contrast to Western medicine, traditional Chinese medicine has certain advantages in the prevention and treatment of many human diseases. In this study, we demonstrated that EJD was able to improve the general conditions of the rats with precancerous liver lesions, as revealed by body weight and improved liver function. Moreover, EJD was sufficient to reverse the precancerous lesion formation induced by DEN and reduced expression of several tumor indicators, suggesting that EJD may suppress liver precancerous lesion development. Notably, the anti-tumor effect of EJD was comparable to that of positive control drug cinobufotalin.

EJD is composed of six traditional Chinese herbs, some of which have been shown to have anti-inflammatory, liver protective, memory improvement, and antitumor effects. EJD is a mixture of six herbs, therefore, it is difficult to dissect the exact roles of these herbs in the protective effects against liver precancerous lesion. In fact, a mixture medication might be more beneficial, more conciliatory, and more balanceable in disease treatment. *Atractylodis Rhizoma* is a dried rhizome of *Atractylodeslancea* (Thunb.) DC or *Atractylodeslancea* (DC.) Koidz. The main components of *Atractylodis Rhizoma* are Atractylolide I, Atractylol III, Atractylol, α-Curcumene, atractylone, β-eucalyptol, and atractylfuran 25. Pharmacological studies have found that *Atractylodis Rhizoma* and its extracts and active ingredients have the ability to interfere with BMP signaling in endothelial cells, downregulate Runx2 activation, and inhibit MMP expression and VEGF secretion, thereby inhibiting tumor formation 26. Moreover, *Atractylodis Rhizoma* can activate the mitochondria-mediated apoptosis pathway, thereby exerting anti-tumor effect 27. The main components of *Atractylodes macrocephala Koidz.* include lactones, glycosides, polysaccharides, and amino acids. Pharmacological studies have revealed that *Atractylodes macrocephala Koidz.* exhibits many roles in the gastrointestinal system, urinary system, cardiovascular system, immune system, reproductive system, and nervous system. It has anti-tumor properties and repairs gastric mucosa. In cancers, *Atractylodes macrocephala Koidz.* has been demonstrated to play a cytotoxic and anti-tumor effect by arresting tumor cells in the S phase 28, 29. *Scutellaria barbarta D.Don* contains a variety of chemical components, including flavonoids, diterpenoids, alkaloids, steroids, and polysaccharides. Many pharmacological effects of *Scutellaria barbata*, such as anticancer, antimutation, anti-inflammatory, antioxidation, immune enhancement, anti-atherosclerosis, and liver protective effects, have been documented 30. The extract of *Scutellaria barbata* has been shown to inhibit the growth and induce apoptosis of liver cancer H22 cells *in vitro* and *in vivo* 31. Additionally, *Scutellaria barbarta* has a certain inhibitory effect on the formation of experimental liver cancer induced by DEN in rats, and can improve various indicators of liver function 32. The main chemical constituents of *Hedyotis diffusa Willd* are anthraquinones and cyclic allene terpenoids 33. *Hedyotis diffusa* and its extracts have multiple anti-tumor effects in human cancers, including leukemia, liver cancer, glioblastoma, lung cancer, colon cancer, breast cancer, bladder cancer, and prostate cancer 34; furthermore, anti-inflammatory, antibacterial, antioxidant, anti-aging, and neuroprotective effects of *Hedyotis diffusa* have been reported. Emerging evidence has demonstrated that *Radix salviae miltiorrhizae* and its active ingredients have anti-inflammatory, antimyocardial ischemia, anti-arrhythmia, anti-platelet aggregation, anti-atherosclerosis, and anti-tumor effects; importantly, *Salviae miltiorrhizae* has anti-liver damage, anti-hepatic steatosis, anti-fibrosis, anti-cirrhosis and anti-HCC effects 35, 36. *Carapax Trionycis* contains animal gum, keratin, iodine, vitamin D, and other ingredients. *Carapax Trionycis* and its active ingredients can inhibit the TGF-β1-induced proliferation of HSC-T6 cells to exert anti-fibrotic effects 37. Moreover, a new heptapeptide from Carapax trionycis is beneficial in carbon tetrachloride-induced acute liver injury in mice 38. These findings indicated a close relationship between EJD in the regulation of liver disease, especially HCC. Indeed, we have provided evidence that EJD is protective against DEN-induced precancerous liver lesions, and this effect seems to be due to the activation of the Hippo signaling pathway. However, the detailed mechanism by which EJD inhibits liver cancer initiation warrants further investigation.

There are several limitations of our study that should be mentioned. Firstly, the exact functional compounds were not investigated and future biochemical studies are warranted. Secondly, EJD alone without DEN treatment should be added to generate more important readouts. Finally, the optimal in vivo dose of EJD has still not been completely explored.

In conclusion, our study reveals an inhibitory effect of EJD in liver cancer initiation and determines the potential mechanism by which EJD ameliorates the symptoms of precancerous liver lesions in a DEN-induced rat model. Importantly, our findings support the possible application of EJD as a potential therapeutic natural product for the treatment of liver cancer.

## Figures and Tables

**Table 1:** Primers used in this study

Gene	Forward primer (5'-3')	Reverse primer (5'-3')
CK19	ACAGAGCGCCAGAACCA	AGGGTAGGAGGCCAGGA
c-Myc	AAGCCACCGCCTACATC	CCTCACTTCCGGTCAGTTT
YAP	ATGCTCTCCCAACTGAACG	GTCATGGCTTGCTCCCA
TAZ	CCGGGCAGAAAACAAGT	GGCAAAGCCTATCTTCCAG
GAPDH	ATGGCTACAGCAACAGGGT	TTATGGGGTCTGGGATGG
